# Stoicism, the physician, and care of medical outliers

**DOI:** 10.1186/1472-6939-5-8

**Published:** 2004-12-09

**Authors:** Thomas J Papadimos

**Affiliations:** 1 Department of Anesthesiology, Medical College of Ohio, 3000 Arlington Avenue, Toledo, Ohio 43614, USA

## Abstract

**Background:**

Medical outliers present a medical, psychological, social, and economic challenge to the physicians who care for them. The determinism of Stoic thought is explored as an intellectual basis for the pursuit of a correct mental attitude that will provide aid and comfort to physicians who care for medical outliers, thus fostering continued physician engagement in their care.

**Discussion:**

The Stoic topics of good, the preferable, the morally indifferent, living consistently, and appropriate actions are reviewed. Furthermore, Zeno's cardinal virtues of Justice, Temperance, Bravery, and Wisdom are addressed, as are the Stoic passions of fear, lust, mental pain, and mental pleasure. These concepts must be understood by physicians if they are to comprehend and accept the Stoic view as it relates to having the proper attitude when caring for those with long-term and/or costly illnesses.

**Summary:**

Practicing physicians, especially those that are hospital based, and most assuredly those practicing critical care medicine, will be emotionally challenged by the medical outlier. A Stoic approach to such a social and psychological burden may be of benefit.

## Background

Medical outliers are defined in health care reimbursement, especially in the prospective payment system, as those patients who require an unusually long hospital stay or whose stay generates unusually high costs, i.e., the most severely ill [1]. According to Meadow et al., racial and Hispanic minorities are more likely to be outliers, as are urban dwellers, and those who live in counties in the USA that have poverty rates greater than 16.7% [2]. The ranks of medically uninsured in the USA have risen to the highest level since 1998 (45,000,000) while at the same time an additional 1.3 million people have fallen below the poverty line [3].

Financial concerns have overwhelmed the American medical educational system and hospitals [4,5]. Medical outliers have become a significant problem. The US Congress has developed a system of payments through Medicare to protect hospitals from high cost patient stays. Teaching hospitals and smaller public hospitals have a higher percentage of outliers. These hospitals are frequently subjected to patient "dumping" by larger, more powerful hospital systems trying to rid themselves of less lucrative clientele [6]. To make matters worse, teaching and public institutions may have their elective surgery schedules (normally profitable) impacted by the medical outlier problem [7,8].

Eric Cassell has pointed out that medical practice is now being shaped by commercial considerations; the issue of financing health care dominates all facets of medicine, including education, research, the relationships between physicians, and the relationships between physicians and their patients [9]. Nonetheless, "sick people are often cared for by physicians who, though burdened by the system in which they work, are dedicated to the sick and to medicine. Doctors who love their profession and who devote their lives to it are not rare" [9]. However, caring for medical outliers can be a burden for even the most dedicated physicians.

In a previous debate an argument was made, using the works of Kant and Hegel, that physicians have an obligation to treat medical outliers [10]. Here an argument is made using Stoic thought in the pursuit of a corollary to support that maxim (obligatory care of medical outliers by physicians). This corollary, that a physician's mental state (attitude), will determine his comfort in pursuing the above maxim, is supported by the Stoic view of determinism.

Stoics will argue that becoming a physician is predetermined by Nature, or God, and, that having occurred, it must also be predetermined that a physician will care for medical outliers. Thus, the only thing physicians can alter, or control, is their attitude. Zeno, the founder of Stoicism, explains,

"A man's excellence or virtue does not depend on his success in obtaining anything in the external world; it depends entirely on having the right mental attitude toward things" [11].

Stoicism teaches that the universe is rational, that it can be explained rationally, and organized rationally. Stoics taught that **logos**, the ability of humans to think, plan, and express themselves, was inherent in the cosmos. Therefore, logos is part of Nature, or God,

"God and man are related to each other at the heart of their being rational agents. If a man fully recognizes the implications of this relationship, he will act in a manner which wholly accords with human rationality at its best, the excellence of which is guaranteed by its willing agreement with nature. This is what it is to be wise...." [12].

Stoics believed that a cosmic thread related every event to another and that such a premise allows a human to live a life at one with Nature, or God. In acquiring such an understanding a human could strike an accord between his or her attitudes, actions, and the course of events.

The reason for having a good mental attitude and the understanding that a cosmic thread relates all actions and events, according to the Stoics, is that God rules all and nothing can occur unless God wills it. Accepting everything that happens to one's self will bring contentment,

"So the good man will accept everything, knowing that it is not only unalterable, since Fate determines all, but also the work of God, the perfect being; namely that his happiness depends entirely upon himself, and not at the mercy of other persons or the play of outside forces. What brings happiness is to have the right attitude, to choose the right actions, to aim correctly at the mark" [11].

Therefore, to "try" is within a man's power, and good intentions are indeed enough according to Stoic philosophy. The ability to succeed is not necessarily within man's grasp, but his attitude is his own doing. If a person does his or her best and has no self-reproach for this effort, then he or she is one with Nature, or God.

There are difficulties with accepting Stoic philosophy, which will be reviewed later. Nonetheless, the Stoic view of a physician's approach to the care of the medical outlier will be advocated as one that may allow a physician to be successful in outlier care. Success does not refer to monetary remuneration, but an approach that will allow a physician mental comfort (a good attitude) in such an advocacy.

Physicians must understand the concepts of good, the preferable, the morally indifferent, living consistently, appropriate actions, the cardinal virtues, and the passions to successfully apply the Stoic view.

## Discussion

### The good, the preferable, and the morally indifferent (in "right actions")

What is good? Any physician pondering this question may come up with answers such as a lucrative medical practice, minimal on-call days, or passing medical board examinations. These things are good, especially when compared to poverty, sickness, or unemployment. However, the Stoics believed what was "good", was also morally perfect (virtue, virtuous acts and virtuous people). Virtue and virtuous things belonged in a league of their own. If you were virtuous, according to the Stoics, you were "good", therefore happy, and this was moral perfection. If you were virtuous you always did what was morally right. Things that are "bad" are morally imperfect (not virtuous); here we speak of evil and wickedness, not poverty, illness, or death.

The Stoics' view of good and bad were extremes of perfection and imperfection. Beauty, wealth, a good job, and a good marriage were things that were preferable, but not morally "good". Illness, poverty, and death were less preferable, but not morally "bad". However, neither the preferable, nor the less preferable were considered "good" or "evil"; they were considered morally indifferent,

"Goodness, however, and knowledge, although they had value of a unique kind, could not be the only things to have value. *Right action *(author's italics) is a matter of choice concerned with morally indifferent things – will you look for wealth or accept poverty, marry or remain a bachelor, live or die? – and choice between absolutely different alternatives would not involve knowledge or reason.... Virtue then can consist in the effort to obtain these things that have value and avoid their contraries, and knowledge can be knowledge of what is to be preferred. But since things of this sort are not "good" or "bad", it is of no importance whether one has them or does not have them, as far as goodness is concerned. The good intention is enough; achievement may be impeded by forces outside a man's control" [11].

It can generally be agreed upon that caring for medical outliers, ideally, is a "right action" because we are dealing with sickness and economics, i.e., morally indifferent things that are not preferable. Many times caring for medical outliers is something we must do to keep our position, fulfill our contract, or to avoid a lawsuit. However, if we speak of doing what is right in the virtuous, or morally perfect sense, caring for medical outliers must be more than moral indifference. It must be an act of virtue, but can only be so if the physician is doing it out of the deepest sense of duty. Virtue in this case means having the right mental attitude toward outlier care and understanding that it is more than an ordinary good, it is an action that stands morally in a class of its own.

### Living consistently

Philosophers in ancient Greece would inquire, "What is the goal of a perfect life" [11]. The Stoic would answer, "living consistently". It means to live harmoniously because those who live in conflict are unhappy.

Zeno explained that "the single plan by which life should be lived must be a plan formed by correct reason, and this would be one that is natural in the sense that it accords both with man's nature and with universal nature" [11]. In advocating the position of the Stoics it is very important to understand that they believed that man inherently considered the interests of his fellow humans important and accepted whatever difficulties divine providence placed upon him so that the wider plan of nature, or God, could be implemented.

To be happy or content in the care of medical outliers a physician must have this consistency of life and an absence of conflict in this regard. To be able to understand this concept and accept it, the physician must be aware that he or she is a part of the whole. For example, let us say a flower flourishes in a garden; we understand what "to flourish" means. Also, let us say we observe a group of birds, some are healthy and some are not. Therefore, we know what the natural condition, or norm, for a flower and a bird should be. This norm is good. So each thing, flower, or bird has a good that is a universal. There is a universal condition or nature that is appropriate for each thing,

"Eating hay is natural to horses, but not to men. It accords with universal nature that horses should eat hay and that men should speak a language. But the former is inappropriate to men and the latter to horses. Universal Nature sanctions a norm for particular things – the nature of plants, animals and men – by reference to which they can be said to attain or not to attain their individual ends" [12].

Thus we can understand what is meant by "the part". Man is born and man will die. Man fights the Universal Nature of death, a particular "norm" of humans. In struggling against his role, or his "part" in nature, man may do extraordinary things to keep himself healthy or alive. A man may decide to preserve his home or his child's education by not having (paying for) health insurance. A physician, for example, may decide that a patient needs an organ transplant. The physician will engage this struggle whether or not the patient has health insurance, and the end result may make the patient a medical outlier, physiologically and/or economically. Universal Nature may sanction a norm for particular things, but humans, and especially physicians, often struggle against their role as a "part" and come into conflict with nature.

Each flower, bird, person, or physician is a part of Nature's whole. Contrary events may happen to an individual "part". Birds are hunted and eaten, flowers are cut and put into a vase, and severely ill humans may end up on long-term ventilatory support. The Stoics believed that such events are part of nature's order. Such a view may be contrary to a human observing the whole of nature. However, if the human perspective is removed from an event then, "From the perspective of the whole even such conditions are not unnatural, because all natural events contribute to the universal well-being" [11].

The Stoics combine their views of the part and the whole. They view the whole as perfect and the nature of perfect requires inequities and incompatibilities; nothing that happens to a human is disadvantageous to him or her, nor is it a disadvantage to nature. Nature is perfect, so according to Stoicism, suffering does not occur for its own sake, but "it is necessary to the economy of the whole" [12].

For physicians to live consistently they must understand their place in nature. Stoics would explain to today's physician that their contentment in dealing with medical outliers depends on this understanding of nature. If this is understood and accepted there will be no conflict and "living consistently" will be attainable.

### Appropriate actions

Most physicians hope to make the right, or moral, decisions in regard to their actions. Caring for medical outliers is a choice that has to be made by many physicians. Stoics believed it was always appropriate to act virtuously, but acting virtuously can only occur when a man is "perfectly good", and since men are not "perfectly good" then physicians cannot act virtuously, in other words, be morally perfect. However, Stoicism has made a niche for appropriate actions, "to act virtuously is always morally good, and to act faultily is always bad, to act *appropriately *is not in itself either good or bad in the sense of being morally "good" or "bad" [11]. Even though physicians as humans are not perfect, and thus cannot act virtuously, nonetheless they can make appropriate decisions, take appropriate actions, and "do the right thing".

A physician may care for a medical outlier, but it is not necessarily a morally good action if he or she does it without the complete understanding of why it is the right thing to do. In other words, taking care of a medical outlier is a just action, and thus an appropriately good action, but only if the physician is doing it without duress or not being mandated to attend to the patient (such as being on-call, by contract, or even being shamed into doing it).

### Cardinal virtues

Irrational forces plague a man's mind according to Plato, and these forces have to be controlled before a man could have the needed knowledge to act virtuously. The Stoics, however, did not feel this was true. They felt that if a man could be trained to think correctly, then he could learn to act virtuously.

Zeno went on to define four cardinal virtues that were necessary for a man to acquire to be successfully trained to think correctly so that he could act virtuously. He used Plato's work as a basis and he defined the four virtues in terms of the fourth virtue, wisdom. **Justice **was wisdom concerned with distribution. **Temperance **(self-control) was wisdom concerned with acquisition. **Bravery **was wisdom concerned with endurance. **Wisdom **was defined as "knowledge of what should and should not be done, or knowledge of what is good or bad or neither" [11].

Medical outliers require justice. Physicians must be able to distribute their actions (medical practice) fairly and equitably to all those who are in need of such services. Temperance, or self-control, must be learned or acquired. Physicians have to control their emotions when assigned to a medical outlier, having their patients turn into medical outliers, or seeing other parties' responses to medical outliers. Physicians should allow neither anger, frustration, anxiety, nor fear to overtake them. Enduring the endless days, weeks, or months of caring for a medical outlier certainly requires bravery and stamina. Fielding the unending phone calls and the constant re-tuning of a patient's hemodynamic status can be of marathon proportions. Wisdom is what should or should not be done and what is good or bad must not only apply to the type and amount of medical care, but it should also encompass the virtue of the acts of justice, compassion, and care given to the medical outlier.

### The passions

Physicians need emotion. The Stoics did not disagree, but they wished to eliminate passion (**pathos**) or what many of them called a mental disturbance. The Stoic "passion" is defined as an excessive uncontrollable drive due to an overestimation of the worth of the "indifferent" things (or events) mentioned previously. Nonetheless Stoics taught that to have great affection was indeed desirable, but at the same time one should remain passionless. Animals are driven to an action because of a stimulus, but in man such a stimulus (or impulse) requires the mind to accede to the stimulus. The Stoics found this to be important because they felt it to be a point of distinction between humans and animals. To the Stoics all living animals were compelled to respond to stimuli by their psyche, a mix of fire and air that was responsible for the functions of living animals (they held that the psyche was not immaterial and could be physically damaged). There are times, however, when a man's mind is out of control and his passions become excessive [11].

There were four kinds of passion the Stoics recognized: fear, lust, mental pain, and mental pleasure (as opposed to physical pleasure). The passions were explained by F.H. Sanbach,

"Fear is a contraction of the psyche caused by the belief that something bad is impending. It causes paleness, shivering, and thumping of the heart. But the belief is false: what is feared is not what a Stoic calls "bad", but one of the morally indifferent things, e.g. death, pain, ill-repute. Fear is the result of exaggerating their importance, of believing they will bring real harm, whereas they do not affect man's essential moral being and if they come are to be accepted as part of the great plan of nature. Lust is a longing for something believed to be good, but again is falsely so believed, since the supposed good is morally of the psyche. Mental pain is a contraction of the psyche resulting from the belief, again erroneous, that something bad is present.... Pleasure was defined as an irrational expansion of the psyche caused by the supposed presence of something good.... What is thought to be good is not in fact good, but at the best acceptable" [11].

In many instances the passions do come into play when a physician cares for a medical outlier. Fear affects the physician from several perspectives. Physicians fear for adverse outlier outcomes, not only for the patient's sake and that of the family, but also out of concern for potential litigation, non-reimbursement for services rendered, and long hours incurred in the care of the patient.

In regard to lust (desire), something believed to be good, but falsely so, several points can be made. The Stoics spoke of many types, or species, of lust, anger being foremost among them. This species of lust is very appropriate to discuss in regard to care of the medical outlier. Physicians do get angry and occasionally act out when challenged. Outliers involve a large investment of emotion, time, and a potential loss of income on the part of the physician (he or she could be caring for patients who are less involved and whose medical insurance has expired). Also, hostility toward staff for small deviations from the plan of care may occur more frequently than the staff would like. As the patient's course of illness drags on physicians may have anger for the patient and the family (even though it may be well concealed). Such anger occurs because the patient does not improve or improves too slowly. There may also be anger towards the family for asking too many questions or questioning the plan of care the physician is following. In addition, the family may also want other physicians consulted or more time from their current physician. What is, in fact, happening is that the family is merely trying to exert control over what little they can yet control.

Mental pain or anguish is self evident in medical outlier care. The previous passions of fear and lust (the species of anger) contribute to the burden of mental pain. The species of mental pain that Stoics address include grief and pity, two potentially powerful distorters of judgment for physicians.

Mental pleasure in caring for those that are seriously and/or chronically ill is not as self-evident. Again we do not speak here of physical pleasure. The species of mental pleasure include "pleasure at unexpected 'benefits', pleasure at other people's misfortunes, pleasures caused by deceit and magic" [11]. Physicians do not take pleasure in the misfortunes of their patients, but there may be interplay of this element when dealing with their colleagues in regard to medical outliers. There are times when various physicians have differing views as what should be done in the course of a patient's care. When one physician is "wrong" and another is found to be "right" concerning a particular decision, procedure, diagnosis, or course of therapy, there are instances of gloating, or taking pleasure in a colleague's fall, error, or misperception.

There is no doubt that medical outlier care evokes passions. There is little for a physician to do but his or her best in regard to giving health care. Much is out of the physician's control, and therefore much of Stoic thought is applicable; control the passions, keep a good attitude, have an open mind about plans of care, have an open mind as to who can participate in decisions (patient, family, other health care providers), understand what is a reasonable outcome, and remember that is does not matter who gets credit for good outcomes [13].

### Flaws in Stoic thought

If all human events and actions are predetermined how are human freedoms and free will to be addressed? Universal causation is the bedrock of Stoic philosophy. If human attitudes and beliefs are within an individual's power or sphere of influence, is this truly congruent with Stoic determinism?

Robert L. Arrington illustrates the human attitude towards sickness as a foible in Stoic thought [14]. Illness can be a misfortune or an " indifference". The Stoics seem to hint that we should see illness as an "indifference" and a misfortune and then choose. If we apply universal causation in this matter there must be a cause for us to view illness one way or another. Arrington's interpretation of this dilemma in Stoic philosophy is illuminating,

"And if the causes that exist prior to our forming the attitude lead us to perceive the illness as misfortune, *it is not possible for us to perceive it as a matter of indifference*. If, on the contrary, the causes lead us to assume the attitude of indifference, then it becomes impossible for us to see the illness as misfortune. Either one of the sets of courses or the other must exist, from which it follows that it is either impossible for us to feel misfortune or impossible for us to feel indifference. If one of these options is impossible, the attitude we take is necessary in which case we really didn't have any options at all. And without options or choices, there is no thing as freedom or voluntary behavior. And, so it seems, our attitudes and beliefs are not in our power" [14].

This argument regarding whether universal causation and determinism is consistent with a free will has been debated for over 20 centuries. Today there are philosophers on both sides of the issue.

Another flaw is the Stoic approach to evil. Stoics simply tell us it does not exist; events may seem evil, but they are not. Stoics teach that only the human perspective allows the interpretation that evil exists. Religions of the world, many philosophers, and people who have viewed and/or endured suffering cannot agree with the Stoics.

A further distortion in Stoic thought involves the idea that the life of virtue is the only "good" life. What about the "preferred" things that we as humans know make our lives better? What is wrong with "attaining the goals of impulse" [14]? There was a gradual progression in the evolution of later Stoic philosophy to allow the acceptance of the "preferable" things and this erosion of principle led to many attacks on Stoicism from other philosophical quarters.

And, finally, the Stoics felt the universe was rational and in unity. A divine thread ran through the cosmos connecting everything and everybody. Many philosophers cannot accept this concept. However, as we see the progression of this line of reasoning as it regards the study of the "string" theory in physics and the further work and modification of Einstein's views of relativity, we realize that there may be a mathematical basis to existence. The Stoics may be criticized about their "thread" through the cosmos, but when we discuss how time "bends" and describe gravity as "curved space" the critics of Stoicism may be tightrope-walking this same thread.

The health care rendered to medical outliers can be for a substantial length of time and cost to the medical practitioner. The intensive interaction with the patient, his or her family, consultants, nurses, other ancillary staff, and the institution can sap the performance of involved physicians.

Placing the argument for the predetermination of events aside, if one is a physician that is involved in a hospital-based specialty, critical care medicine, or as surgeon, the fact of the matter is that medical outliers will come to your door. By fate or by choice physicians in the above-mentioned areas will be engaged with outliers. As the Stoics point out, the future is coming at you and there is nothing you can do about it, except adjust your attitude.

While engaged in such an endeavor physicians will "try" to do their best, hopefully without self-reproach as to their efforts. Physicians, as humans, cannot be "good", or morally perfect. They become tired, hungry, worry about the bills, their children, their practice, hospital policies, etc. Nonetheless, most physicians realize and understand what appropriate actions are necessary in regard to the most ill and poorest of patients. Also, though physicians may not be morally perfect (virtuous in the Stoic sense), they know what is "preferable" for their patients, i.e., to get well, go home and be with their families. These are morally "indifferent" things, but as Stoics point out, "virtue then can consist in the effort to obtain these things that have value and avoid their contraries, and knowledge can be knowledge of what is to be preferred" [11].

As mentioned previously, physicians frequently struggle against their role as a "part". By the mere fact that physicians acknowledge struggles with or against insurers, patients, families, colleagues, ancillary staff, and institutions there is realization that they are part of a "whole", but at the same to "live consistently" requires an understanding of one's role in nature and the need for absence of conflict. In the struggle to help the critically ill, the chronically ill, or the incredibly poor, avoiding conflict is challenging.

The cardinal virtues of justice, temperance, bravery, and wisdom come with upbringing, education, and life experiences. Lacking the proper intellectual or nurturing environment will not allow the flourishing of these virtues in an individual. In schools of medicine the faculty attempt to inculcate these virtues in the students, although they are not always successful.

The Accreditation Council for Graduate Medical Education (ACGME) has recently mandated six core competencies for resident education [15]. One of these competencies is Professionalism (and ethics). This, in effect, formalizes ethics education at the graduate level (residency). Such a graduate level discourse should be preceded by a problem-based learning format at the medical school level, preferably before the students begin their clinical work. Thus, the Stoic view, or any other philosophical view or ethical concept, could be taught pre-clinically and then reinforced in an ACGME residency format.

Justice is a far-reaching concept that more physicians need to embrace. It is difficult to teach and is best acquired through experience of its antithesis. Temperance, or self-control, can be mandated by medical staff guidelines and licensing boards, but this virtue actually needs to have been ingrained before medical school. Bravery is something that residency training seeds in a physician through the frequent facing of dying or hostile patients that come through a medical practice, especially at an academic institution that cares for the disenfranchised. Wisdom will come with time and maturity. Both are needed to acquire wisdom. Physicians, medical students, and residents can, indeed, be taught to think correctly, as the Stoics emphasized. However, individuals will have varying degrees of success depending their rearing/nurturing and educational environment.

Emotion is necessary to physicians, but the passions, those uncontrollable mental disturbances due to overestimation of the value of "indifferent" things, may blind them in their judgment and decision-making. It is important that when making important decisions in regard to medical outliers that the passions be "checked".

To avoid frustration, disappointment and unhappiness in the practice of medicine as it regards medical outliers, physicians must do two things: (1) control things that are within their power (attitudes, desires, beliefs), and (2) be indifferent to the things that they cannot control (things external to themselves) [16].

Even though Stoicism has evoked controversy for over twenty centuries it is relevant to a physician who must juggle patients, procedures, therapies, and colleagues in the care of a patient who has maximally taxed medical insurers, institutions, other practitioners, and their own families.

## Summary

Insurers and institutions may have financial burdens, but those providing patient care, especially physicians, bear a disproportionate slice of the mental anguish associated with the care of medical outliers. Here an argument has been made that applying the philosophical tenets of Stoicism to the physician's intellectual pursuit of how to deal mentally with the care of medical outliers is appropriate. Physicians that are hospital based and those practicing critical care medicine may well be the providers most emotionally challenged by outliers. A Stoic approach to such a social and psychological burden may be helpful.

## Competing interests

The author(s) declare that they have no competing interests.

## Author's contributions

TJP is responsible for the manuscript in its entirety.

## Pre-publication history

The pre-publication history for this paper can be accessed here:


